# ﻿Three new species of the sea fan genus *Muricea* (Cnidaria, Octocorallia, Plexauridae) from the northwest region of Mexico

**DOI:** 10.3897/zookeys.1169.89651

**Published:** 2023-07-18

**Authors:** Osvaldo Hernández, Jaime Gómez-Gutiérrez, Carolina Galván-Tirado, Carlos Sánchez

**Affiliations:** 1 Departamento de Plancton y Ecología Marina, Centro Interdisciplinario de Ciencias Marinas, Instituto Politécnico Nacional, Av. IPN, s/n, CP 23096, La Paz, Baja California Sur, Mexico Instituto Politécnico Nacional California Sur Mexico; 2 Departamento de Ciencias Marinas y Costeras, Universidad Autónoma de Baja California Sur, Carretera al sur km 5.5, CP 23080, La Paz, Baja California Sur, Mexico Universidad Autónoma de Baja California Sur California Sur Mexico; 3 Consejo Nacional de Ciencia y Tecnología, Av. Insurgentes Sur 1582, Col. Crédito Constructor, Alcaldía Benito Juárez, C.P. 03940, Ciudad de Mexico, Mexico Consejo Nacional de Ciencia y Tecnología Ciudad de México Mexico

**Keywords:** Baja California, Gulf of California, Malacalcyonacea, mtMutS, species-groups, taxonomy

## Abstract

Twenty-one nominal species of *Muricea* have been reported in the Eastern Pacific with nine of them reported in the Mexican Pacific. We describe three new species of *Muricea: Muriceaambarae***sp. nov.** and *Muriceacacao***sp. nov.**, from rocky reefs on the central and the northern Gulf of California and the Pacific coast of Baja California Sur, and *Muriceamolinai***sp. nov.**, from the Pacific coast of Baja California Sur. *Muriceaambarae***sp. nov.** and *M.cacao***sp. nov.** are taxonomically allied to the nominal species *Muriceafruticosa* Verrill, 1869 due to the morphological similarity of colony growth patterns and the phylogenetic closeness based on the mitochondrial MutS gene (mtMutS); but differ mainly in the calyx form and composition of sclerites. The main morphological differences between the new *Muricea* species are in their sclerite forms and color; *M.ambarae***sp. nov.** has orange-colored colonies, thin leaf spindles and tuberculated blunt spindles, while *M.cacao***sp. nov.** has dark brown colored colonies, strong spinous spindles and an absence of tuberculated blunt spindles. *Muriceamolinai***sp. nov.** is phylogenetically close and morphologically similar to *Muriceasquarrosa* Verrill, 1869 in the growth form of the colony and tubular calyces; but has dark brown colored colonies and has calyces from the base to the branch tips. With these three new species, the total number of *Muricea* species reported in the Mexican northwest region increases to twelve and a total of 24 nominal species in the Eastern Pacific.

## ﻿Introduction

Sea fans of the family Plexauridae of the genus *Muricea* Lamouroux, 1821 currently include 30 nominal species worldwide: nine species are distributed in the Western Atlantic Ocean and 21 species are distributed in the Eastern Pacific (Bayer 1959, 1994; Breedy and Guzman 2015, 2016a, b; McFadden et al. 2022, 2023). Breedy and Guzman (2015, 2016a) subdivided the 21 Eastern Pacific *Muricea* species into four morphological species-groups: 1) *Muriceasquarrosa* species-group, currently including four species characterized by the presence of tubular calyces; 2) *Muriceafruticosa* species-group encompassing five species with shelf-like calyces and unilateral spiny spindles; 3) *Muriceaplantaginea* species-group including four species with a conspicuously elongated lower coenenchyme of the calyces, and foliated or thorny spindles; and 4) *Muriceaaustera* species-group encompassing eight species characterized by candelabrum-like and robust colonies, thick branches and thick coenenchyme.

Nine *Muricea* species have been reported on the Gulf of California and Pacific coast of Mexico: *Muriceaaustera* Verrill, 1869; *Muriceacalifornica* Aurivillius, 1931; *Muriceaechinata* Verrill, 1866; *Muriceaformosa* Verrill, 1869; *Muriceafruticosa* Verrill, 1869; *Muriceahebes* Verrill, 1864; *Muriceaplantaginea* (Valenciennes, 1846); *Muriceapurpurea* Verrill, 1868; and *Muricearobusta* Verrill, 1864 (Verrill 1868, 1869; Breedy and Guzman 2015, 2016a). Here we describe three new *Muricea* species discovered on the northwest Mexican Pacific coast showing distinctive diagnostic morphological features: *Muriceaambarae* sp. nov. and *Muriceacacao* sp. nov. are similar to *M.fruticosa* in calix form, and *Muriceamolinai* sp. nov., which has morphological features similar to *M.squarrosa* Verrill, 1869 mainly in the form of the calyces and coenenchymal sclerites. Internal and external diagnostic morphological characteristics of these three new species were analyzed and compared among several morphologically similar *Muricea* species distributed in the Eastern Pacific. These morphological comparisons were here also explored using evidence from the mitochondrial MutS gene (mtMutS).

## ﻿Materials and methods

### ﻿Field work and morphological comparisons

All sea fan colonies analyzed for the present study were collected by scuba diving (< 50 m depth) during systematic monitoring surveys carried out between 1998 and 2020 in 250 locations in the Gulf of California and along the Pacific coast of Baja California Peninsula, Mexico (Fig. 1A–D). The collected sea fans were preserved dry or in ethanol (96%). A portion of each sea fan colony was macerated in sodium hypochlorite to extract the sclerites, washed several times with distilled water, and preserved in 96% ethanol for further microscopic analyses. Sclerites were air-dried and attached to aluminum stubs with double adhesive bands. They were coated with gold using a sputter coater (Polaron E5100) in a gold atmosphere and observed under a Hitachi S-3000 N scanning electron microscope (SEM) at 20 kV following standard methods (Hernández et al. 2021). All digital images and image plates were edited and prepared using Adobe Photoshop 21.1.3 and Corel-PhotoImpact X3 software. Holotypes and paratypes of the three new species were described based on diagnostic internal and external morphological characters and compared with nominal species closely related within the genus *Muricea* (Breedy and Guzman 2015; 2016 a, b). About 450 colonies of two nominal species of *Muricea* (*M.fruticosa* and *M.plantaginea*) and undescribed species (with specimens of the three new species) are deposited in the collection of the “Programa de Investigación para la Conservación de la Fauna Arrecifal” (PFA) of the Universidad Autónoma de Baja California Sur (UABCS), La Paz, Baja California Sur, Mexico. The external and internal morphological descriptions of the colonies of the three new species of *Muricea* were prepared using standard criteria and nomenclature (Bayer et al. 1983; Calvo and Breedy 2002). All holotypes and paratypes were deposited in the Invertebrate Zoology Collection, National Museum of Natural History, Smithsonian Institution, Washington, DC, USA (**NMNH**). Taxonomical classification was based on McFadden et al. (2022, 2023). Maps showing the toponymy and the sampling locations of the holotypes (type locations) and paratypes of each new *Muricea* species were prepared using Surfer ver. 12 software (Fig. 1A–D).

**Figure l.**
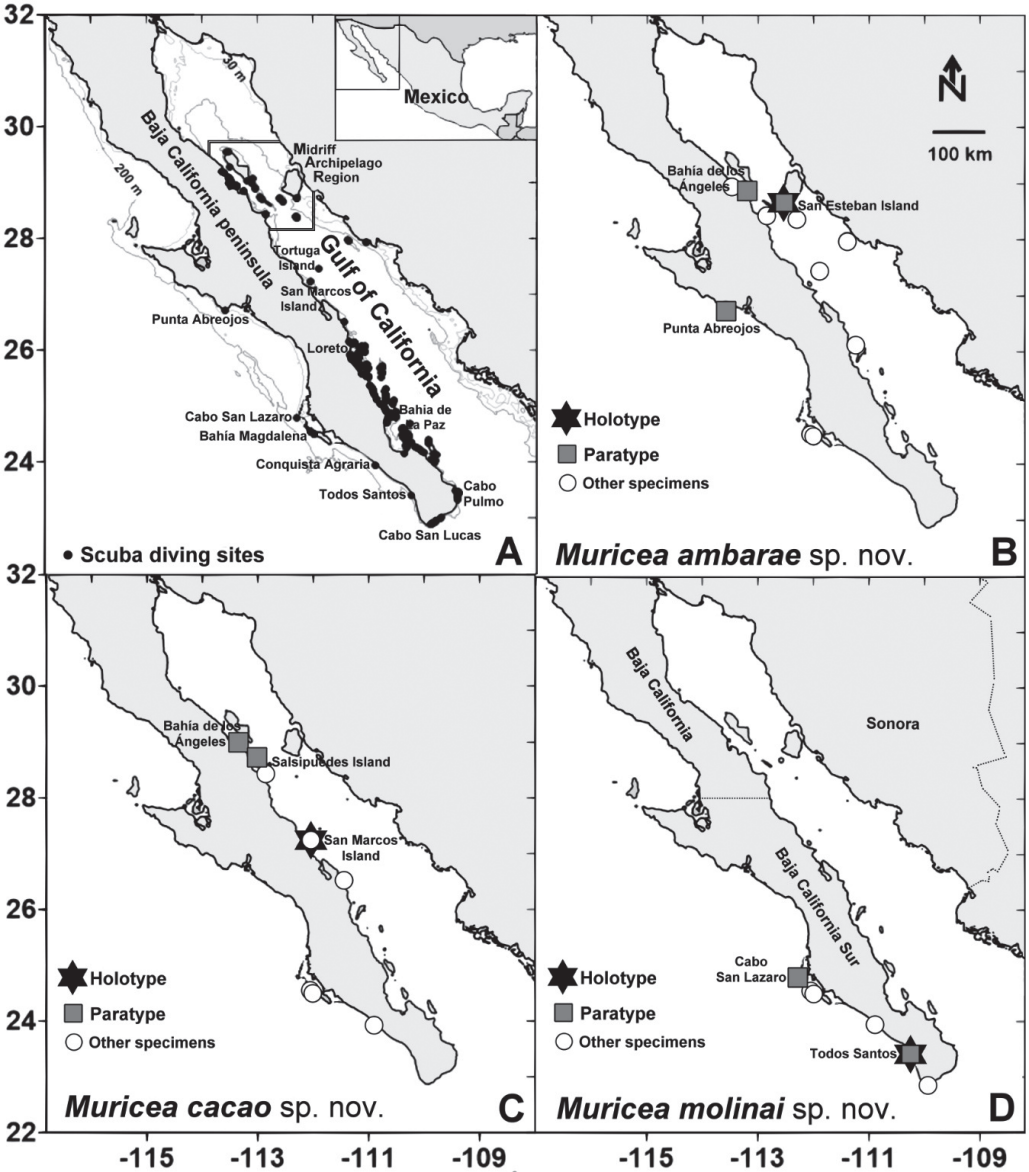
Locations of sample collections of the three new species of the genus *Muricea* discovered along the Pacific coast of Baja California Sur and the Gulf of California, Mexico **A** scuba diving and monitoring census sites **B***Muriceaambarae* sp. nov. collection sites **C***Muriceacacao* sp. nov. collection sites **D***Muriceamolinai* sp. nov. collection sites.

### ﻿Molecular analysis

Sea fan colonies preserved dry were used for molecular analyses and ten sequences (six sequences from the three new species, plus four sequences of nominal species) of a partial fragment (~931 base pair, bp) of the mtMutS gene were generated. The sequences were submitted to GenBank with accession numbers given in Suppl. material 1: data S1. In addition, twelve sequences of *Muricea* reported for the Eastern Pacific Ocean were downloaded from GenBank (https://www.ncbi.nlm.nih.gov/genbank/) for the phylogenetic reconstruction (Suppl. material 1: data S1). All these sequences were aligned and trimmed to 493 bp to have all the sequences at the same length. For detail in the molecular analyses see the Suppl. material 1.

## ﻿Results

### ﻿Systematics

**Phylum Cnidaria Hatschek, 1888**

**Class Anthozoa Ehrenberg, 1834**

**Subclass Octocorallia Haeckel, 1866**

**Order Malacalcyonacea McFadden, van Ofwegen & Quattrini, 2022**

**Family Plexauridae Gray, 1859**

#### ﻿Genus *Muricea* Lamouroux, 1821

##### ﻿ Muricea ambarae sp. nov.

Taxon classificationAnimaliaAlcyonaceaPlexauridae

372FD8CB-5604-5AC0-94CF-4AE886C038D9

https://zoobank.org/4FF4B82B-991F-4E73-BBEC-5520AE772B0A

###### Material examined.

***Holotype*.** USNM 1606629: dry, San Esteban Island (Punta Sureste), Sonora, Mexico (28°40.29228'N, 112°33.24035'W), 20 m depth, 20 °C, 20 June 2010 (Fig. 1B). ***Paratypes*.** USNM 1606630: dry, San Esteban Island (Punta Noroeste), Sonora, Mexico (28°43.22958'N, 112°36.76110'W), 20 m depth, 21 °C, 17 July 2010; USNM 1606631: dry, Bahía de Las Ánimas (Los Choros), Baja California, Mexico (28°50.36868'N, 113°14.80885'W), 18 m depth, 20 °C, 18 July 2010; USNM 1606632: dry, Punta Abreojos, Pacific coast of Baja California Sur, Mexico (26°41.76060'N, 113°34.59960'W), 21 m depth, 16.5 °C, 24 June 1998 (Fig. 1B). The type specimens were collected by Carlos Sánchez.

###### Holotype colony description.

**Figure 2.**
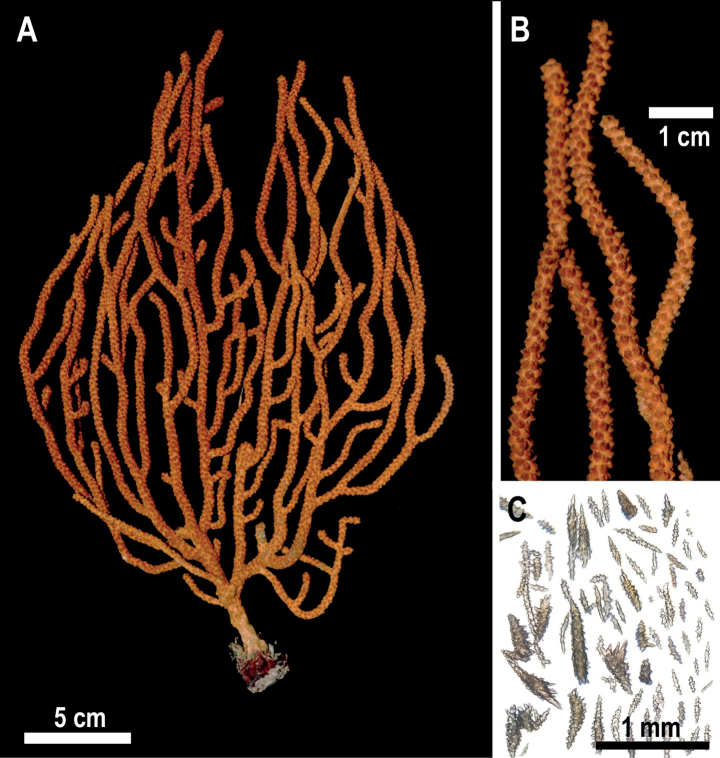
*Muriceaambarae* sp. nov. **A** holotype USNM 1606629 **B** holotype detail of branches **C** axial and coenenchymal sclerites.

Colony with flabellate growth in one plane and laterally branched, 21 cm high and 14.2 cm wide (Fig. 2A, Table 1). The holdfast is an irregular oval, 1.9 cm in length and 1.3 cm wide from which grows the main stem, 5.2 cm in height and 0.5 mm in diameter. There is no coenenchyme on the base of the steam lost during the collect; the base coenenchyme shows a dark gray and brownish bicolored axis. The growth of the branches is lateral and upward. Some terminal branches are short, about 5 mm in length and 3.5 mm in diameter, while the longest ones are 9.5 cm in length and 3.5 mm in diameter (Fig. 2B, Table 1). All the terminal tips are blunt and covered by calyces. Calyces in the colony range up to 1 mm in height and are 1 mm in diameter and are shelf-like in form with an imbricated arrangement. The coenenchyme color is pale yellow, but the coloration of the calyces is reddish-brown, giving the colony an overall pale and dark orange appearance (Fig. 2A, B, Table 1).

**Table 1.** Internal and external characters of *Muriceaambarae* sp. nov., *Muriceacacao* sp. nov. and *Muriceamolinai* sp. nov. with similar *Muricea* nominal species distributed along the Mexican Pacific and Gulf of California collected from 1998–2020 and compared with Breedy and Guzman (2015, 2016a). **Colony growth**: bu = bushy, fa = falling branches, fl = flabellate. **Branching type**: di= dichotomous, irr = irregularly, lb = laterally branched, ob = open branched. **Polyp distribution rows**: im = imbricated, c = close, s = sparsely. **Calyx form**: el= elongated, sl = shelf-like, t = tubular. **Color**: am = amber, br = brownish-red, bi = bicolor, cl = colorless, db = deep brown, dy = dull yellowg = gray, lb = light brown, lo = light orange, o = orange, ro = reddish-orange, py = pale yellow, r = red, rb = reddish-brown, y = yellow, w = white. **Sclerites**: ae = acute end spindles, bs = branched spindles, be = bend spindles, cs = curved spindles, cl= club-like, de = dull ends spindles, lb = lobed, ls = leaf spindles, ps = prickly spindles, r = rods, slr = star-like radiates, str = straight spindles, tbr = tuberculated rods, tbs = tuberculated spindles, uss = unilateral spinous spindles, ws = warty spindles, wr = warty rods.

Species	Colony growth	Branching type	Terminal branches length (cm)	Polyp distribution rows	Pseudoanastomosis	Calyx height elevation (mm)	Calyx form	Colony Color	Outer coenenchymal and calyx dominant spindles	Coenenchymal and calycular spindles maximum size (mm)	Inner coenenchymal spindles	Anthocodial sclerites	Sclerites color
*M.californica*	bu	irr, lb	2.8	c, im	no	1.9	el	ro	ls	0.5	slr, ws	lb, wr	am, lo, ro, py
*M.echinata*	bu	irr, lb	6–3	c	no	2.8–3	sl	rb	uss	2.4	ws	r, bs	o, lb
*M.fruticosa*	bu	irr	1.5–4	c	no	1–1.2	sl	rb, w, bi	uss	2	ws	ws, wr, bs	w, rb, py
*M.galapagensis*	fa	ob	8	s	no	0.6–1	sl	lo	uss	4.1	ws	r, ps	am, lo
*M.plantaginea*	fl	irr, lb	1–5	c, im	no	0.7–1.2	sl	db/w	ls	1	ws	lb, wr	rb, am
*M.squarrosa*	fl	di	4	c	no	2.6	t	lb	cl, cs	1.3	tbs	cl	br, cl, py, y
*M.ambarae* sp. nov.	fl	lb	9.5	im	no	1	sl	o	ls, tbs	1.2	ws	lb	cl, o, y, py
*M.cacao* sp. nov.	fl	lb	9	im	yes	1	sl	br-r	ls	1.7	ae/de-ws	tbr-ae	db, rb
*M.molinai* sp. nov.	fl	lb	8.7	im	no	3	t	g	uss, tbs	2.5	str, cs	tbr, lr	g, am, cl

###### Holotype sclerites.

**Figure 3.**
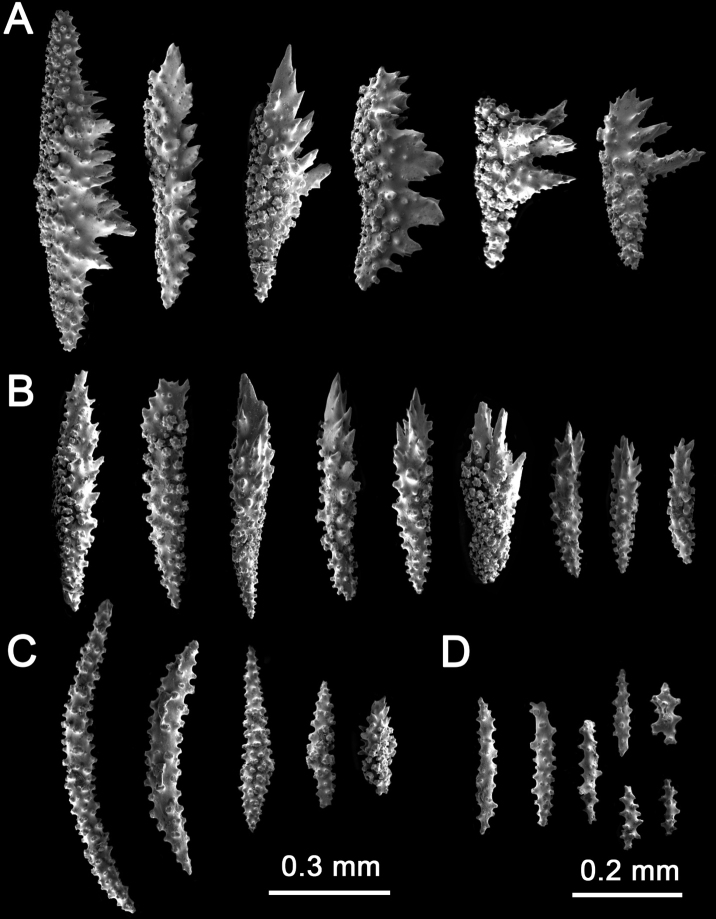
*Muriceaambarae* sp. nov. SEM**A, B** calycular and coenenchymal sclerites **C** axial sclerites **D** anthocodial sclerites.

The sclerites of the outer coenenchyme and calycular are pale yellow or pale orange leaf spindles (0.3–1.2 mm length), tuberculated spindles with blunt ends (0.2–1.1 mm length), and tuberculated spindles with acute ends (0.2–1.1 mm length) (Figs 2C, 3A–D, Table 1). The leaf spindles are more common all around the polyp aperture, and the spindles with acute ends are the dominant type of sclerite in the rest of the coenenchyme (Fig. 3A). The axial sheath comprises thin spindles with acute ends and clubs (0.3–0.9 mm in length) (Fig. 3B, C). These spindles forms have different sizes of tubercles; about 70% of the coenenchyme and calyx sclerites are colorless (Fig. 2C), with the rest of the sclerites pale yellow or pale orange. Anthocodial sclerites are colorless warty rods, 0.2–0.3 mm in length with acute or dull ends (Fig. 3D).

###### Morphological variation.

All 14 colonies of *Muriceaambarae* sp. nov. examined are morphologically like the holotype in colony growth and sclerite form with colony size range observed *in situ* between 4 and 32 cm height (Suppl. material 1: fig. S1A–D). *Muriceaambarae* sp. nov. colonies show differences in color intensity ranging from darker to lighter orange (Suppl. material 1: fig. S1A–D). Only three of these 15 analyzed colonies showed a lax and bushy colony growth form. Qualitative *in situ* observations showed a dominance of the planar form over the bushy form (Fig. 8A, B). The polyps are colorless or white in live colonies (Fig. 8B; Suppl. material 1: fig. S2B, D).

###### Habitat and distribution.

*Muriceaambarae* sp. nov. was collected at two locations, the northern and central regions of the Gulf of California (where the species is more frequently collected), and Bahía Magdalena and Punta Abreojos located along the Pacific coast of Baja California Sur, Mexico (Fig. 1B). *Muriceaambarae* sp. nov. was mostly observed and collected in the Gulf of California on rocky reefs at < 30 m depth and it is currently unknown if this species is present in deeper waters. *Muriceaambarae* sp. nov. shares habitat with *Muriceaplantaginea*, *Muriceafruticosa*, *Muriceaaustera*, *Muriceacacao* sp. nov., *Leptogorgiaalba* (Duchassaing & Michelotti, 1864), and *Ellisellalimbaughi* Bayer & Deichmann, 1960. Colonies of *M.ambarae* sp. nov. distributed in the region of Bahía Magdalena grow on rocky reefs, pebbled seafloors between 5–20 m depth, and in forest-like reefs formed by the brown seaweed *Eiseniaarborea* J.E. Areschoug, 1876 (1–2 m in height) that typically cover large seafloor areas inside the bay. *Muriceaambarae* sp. nov. shares habitat in Bahía Magdalena with *Leptogorgiadiffusa* (Verrill, 1868), *Muriceacacao* sp. nov., *M.molinai* sp. nov., *M.plantaginea*, *M.fruticosa*, and *Psammogorgiateres* Verrill, 1868.

###### Remarks.

**Figure 4.**
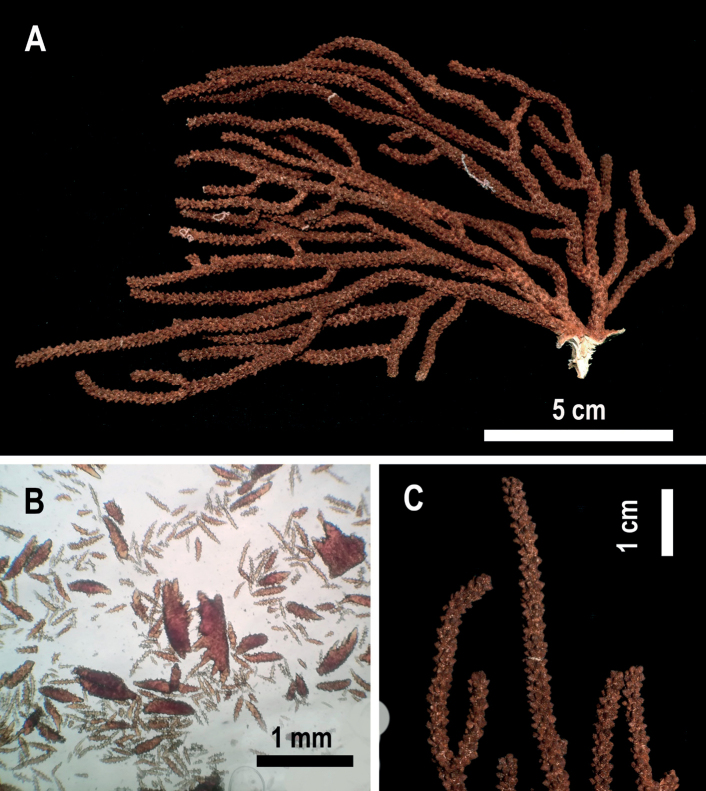
*Muriceacacao* sp. nov. **A** holotype USNM 1606633 **B** anthocodial and coenenchymal sclerites **C** holotype detail of branches.

*Muriceaambarae* sp. nov. (Fig. 2A, B) is similar to *Muriceacacao* sp. nov. in colony growth form patterns and calyx form (Fig. 4A, C), but dissimilar in dichotomous branching, lack of pseudoanastomosis, colony coloration, and sclerite appearance (Table 1). *Muriceaambarae* sp. nov. is also close to *Muriceafruticosa* in the shelf-like form of the calyces, but morphologically differs in colony growth and sclerite composition (Table 1). *Muriceafruticosa* has a bushy growth pattern, irregular branching and its coenenchyme has unilateral spinous spindles. In contrast, *M.ambarae* sp. nov. has a planar colony growth, lateral branching, a single chromotype, and leaf spindles (Table 1), and *Muriceaambarae* sp. nov. has leaf spindles, which are absent in *M.fruticosa*. Thus, we propose to include *Muriceaambarae* sp. nov. in the *M.fruticosa* species-group erected by Breedy and Guzman (2016a). *Muriceaambarae* sp. nov. is also close to *Muriceagalapagensis* Deichmann, 1941, sharing with that species low shelf-like calyces that spread outward, orange colony coloration, and planar colony growth (Table 1). However, *M.galapagensis*, like that of *M.fruticosa*, has falling branches, unilateral spinous spindles, but lacks leaf spindles (Table 1). *Muriceacalifornica* (Suppl. material 1: fig. S3F–I) is morphologically and biogeographically similar to *M.ambarae* sp. nov. (Suppl. material 1: figs S1A–D, S3A–E). However, *M.californica* has high variability in colony growth and coloration and its main population densities occurs in California while *M.ambarae* sp. nov. is mostly distributed in the northern region Gulf of California and does not show evident morphological variability (Suppl. material 1: fig. S1A–D). We conclude *M.californica* and *M.ambarae* sp. nov. are distinct species because they show clear differences in calix form and size, branch diameter and sclerites forms. *Muriceaambarae* sp. nov. have shelf-like slightly raised calyces, terminal branches of up to 3.5 mm diameter, and an absence of torch spindles in the coenenchyme (Suppl. material 1: fig. S3A–E), while *M.californica* has prominent and elongated calyces (almost cylindrical), wider branches of up to 0.5 mm thick, and the presence of torch spindles (Suppl. material 1: fig. S3F–I) (Horvath 2019).

###### Etymology.

The word “*ambarae*” means “amber”, a hard, transparent, fossilized resin produced by some trees. Amber has colorations from pale yellow/orange to a dark orange, like the coloration observed in living colonies of *Muriceaambarae* sp. nov. Mexican amber, also known as Chiapas Amber, dates from 15 to 23 million years old. Since the time of the Mayan culture, its people have believed amber to have healing and protective qualities. The species name is also inspired from the name of the daughter (Ámbar) of Carlos Sánchez.

##### ﻿ Muricea cacao sp. nov.

Taxon classificationAnimaliaAlcyonaceaPlexauridae

1C16B3A1-6247-56F5-AECC-23AD94F60F8F

https://zoobank.org/F91F41A5-DAA5-4D47-9E62-812EFDAFC7D6

###### Material examined.

***Holotype*.** USNM 1606633: dry, San Marcos Island (El Faro-Lobera), Baja California Sur, Mexico (27°15.95706'N, 112°5.51208'W), 15 m depth, 23 °C, 14 July 2010 (Fig. 1C). ***Paratypes*.** USNM 1606634: dry, San Marcos Island (El Faro-Lobera), Baja California Sur, Mexico (27°15.95706'N, 112°5.51208'W), 15 m depth, 23 °C, 14 July 2010; USNM 1606635: dry, Salsipuedes Island (Caleta Falsa Norte), Baja California, Mexico (28°43.78506'N, 112°57.88512'W), 16 m depth, 23 °C, 7 November 1999; USNM 1606636: dry, Bahía de los Ángeles (Punta Pescador), Baja California, Mexico, (28°56.12382'N, 113°22.91976'W), 15 m depth, 20 °C, 19 July 2010 (Fig. 1C). The type specimens were collected by Carlos Sánchez.

###### Holotype colony description.

The holotype is a brownish-red colony, growing flabellate upwards in one plane and laterally branched, 15.8 cm tall and 8.7 cm wide (Fig. 4A, C, Table 1). Polyp rows distribute imbricated (Table 1). The holdfast is 2 cm length and 1 cm wide with a cream-white color in the area without coenenchyme (lost during collect) (Fig. 4A). Two main branches arise from the base; one is 1.4 cm in length and 4 mm in width, and the other one is 1.3 cm in length and 6 mm wide. They are subdivided into three secondary branches, two of them partially fused (pseudoanastomosis) (Fig. 4A). Several terminal twigs are short (1 cm in length), with several reaching 9 cm in height. In both branches, the average diameter is 3 mm, with blunt ends (Fig. 4C). Calyces are shelf-like, 1 mm in height with a diameter of 1 mm, situated all around the branches in an imbricated arrangement. Calyces are acute in the upper part of the colony or on terminal twigs and blunt at the base.

###### Holotype sclerites.

**Figure 5.**
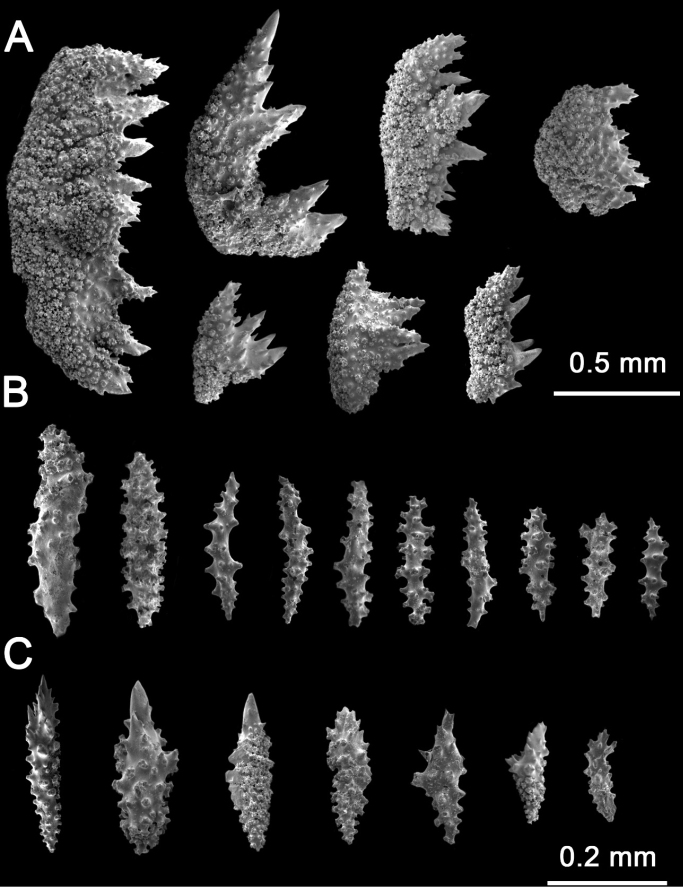
*Muriceacacao* sp. nov. SEM**A, B** calycular and coenenchymal sclerites **C** axial sclerites.

The sclerites of the outer coenenchyme and calyces are dark brown or reddish-brown (Fig. 4B, Table 1). In the outer coenenchymal leaf spindles with a very strongly thorny appearance 0.4–1.7 mm length are dominant (Fig. 5A); sclerites are also tuberculated spindles with acute ends (1.4 mm in length) (Fig. 5B). The inner coenenchymal sclerites (0.18–0.35 mm length), are thin spindles with blunt or acute ends, most of them colorless (80%), while the rest are pale amber (Figs 4B, 5C).

###### Morphological variation.

All the *Muriceacacao* sp. nov. specimens collected and observed *in situ* are morphologically consistent with the macro- and micro-morphology of both the holotype (Fig. 4A–C) and paratypes with colony size range observed *in situ* between 2 and 55 cm height (Suppl. material 1: fig. S2E–F). The main differences among *Muriceacacao* sp. nov. colonies are the growing angle of the branches, the morphology likely influenced by the local water current pattern and available sea floor space on which they grow (Fig. 8C, D). The polyps in living colonies have white neck and colorless tentacles (Fig. 8D; Suppl. material 1: fig. S2F).

###### Habitat and distribution.

*Muriceacacao* sp. nov. is present in the northern part of the Gulf of California from the Santa Rosalía region to the Midriff Archipelago Region (MAR) and along the Pacific coast of Baja California Sur, between Todos Santos and Bahía Magdalena (Fig. 1A, C). *Muriceacacao* sp. nov. (Fig. 1C) and *Muriceaambarae* sp. nov. (Fig. 1B) share similar distribution and habitat.

###### Remarks.

Paratypes of *Muriceacacao* sp. nov. have low morphological variability and the main difference is the width of the branches (Suppl. material 1: fig. S4A–C). *Muriceacacao* sp. nov. (Fig. 4A, B) is similar to *Muriceaambarae* sp. nov. (Fig. 2A, B) in the colony’s growth form; but differs externally because *M.ambarae* sp. nov. always has bicolored colonies present; reddish calyces with an orange coenenchyme and does not have pseudoanastomosis as does *Muriceacacao* sp. nov. In contrast, *M.cacao* sp. nov. has homogenous brown-reddish coloration in calyces and coenenchyme and has pseudoanastomosis (Fig. 8C, D, Table 1). The main morphological differences between both new *Muricea* species are in the sclerite forms present; *M.ambarae* sp. nov. has leaf spindles and the presence of tuberculated, bent spindles (Fig. 3A–D), while *M.cacao* sp. nov. has even more strongly unilateral multi-spinous spindles, and an absence of tuberculated, bent spindles (Fig. 5A–C, Table 1). However, due to the similarities in colony growth pattern and calyx form between *M.cacao* sp. nov. and *M.ambarae* sp. nov. (Table 1), we propose to include both species in the *fruticosa* species-group proposed by Breedy and Guzman (2016a). Even if *Muriceacacao* sp. nov. (Suppl. material 1: fig. S2E–F) is comparable with *Muriceaplantaginea* (Suppl. material 1: fig. S2G–J), the later has prominent and elongated calyces, with elongated lower borders curved inwards (Fig. 9). *Muriceaplantaginea* has large colonies of up to 100 cm height, simple leaf spindles and in Mexican living colonies the polyps are always orange or yellow-orange (Suppl. material 1: fig. S2G–I).

###### Etymology.

The latinized species name “*cacao*” comes from the ancient pre-hispanic Nahuatl “cacao”, which is the seed used in the making of chocolate, but in Latin is “*cacao*”, the species name of the cocoa tree is *Theobromacacao* L. This “chocolate” color is a practical diagnostic characteristic of both preserved colonies and live colonies with retracted polyps, distinguish this species *in situ* from other *Muricea* species.

##### ﻿ Muricea molinai sp. nov.

Taxon classificationAnimaliaAlcyonaceaPlexauridae

1DB7C883-B97A-504A-8DF9-19E50C07604F

https://zoobank.org/F068F6FD-C900-45E7-85B0-55D8BD3355EC

###### Material examined.

***Holotype*.** USNM 1606637: dry, Todos Santos, Punta Lobos (Bajo Fondo del Medio), Pacific coast of Baja California Sur, Mexico (23°21.34806'N, 110°15.40374'W), 35 m depth, 19 °C, 27 August 2016 (Fig. 1D). ***Paratypes*.** USNM 1606638: dry, Todos Santos, Punta Lobos (Bajo Fondo del Medio), Pacific coast of Baja California Sur, Mexico (23°21.34806'N, 110°15.40374'W), 35 m depth, 19 °C, 27 August 2016; USNM 1606639: dry, Bahía Santa María, Cabo San Lázaro (Roca del Cabito), Pacific coast of Baja California Sur, Mexico (24°44.85966'N, 112°15.56952'W), 29 m depth, 22.4 °C, 13 November 2013 (Fig. 1D). The type specimens were collected by Carlos Sánchez.

###### Holotype colony description.

**Figure 6.**
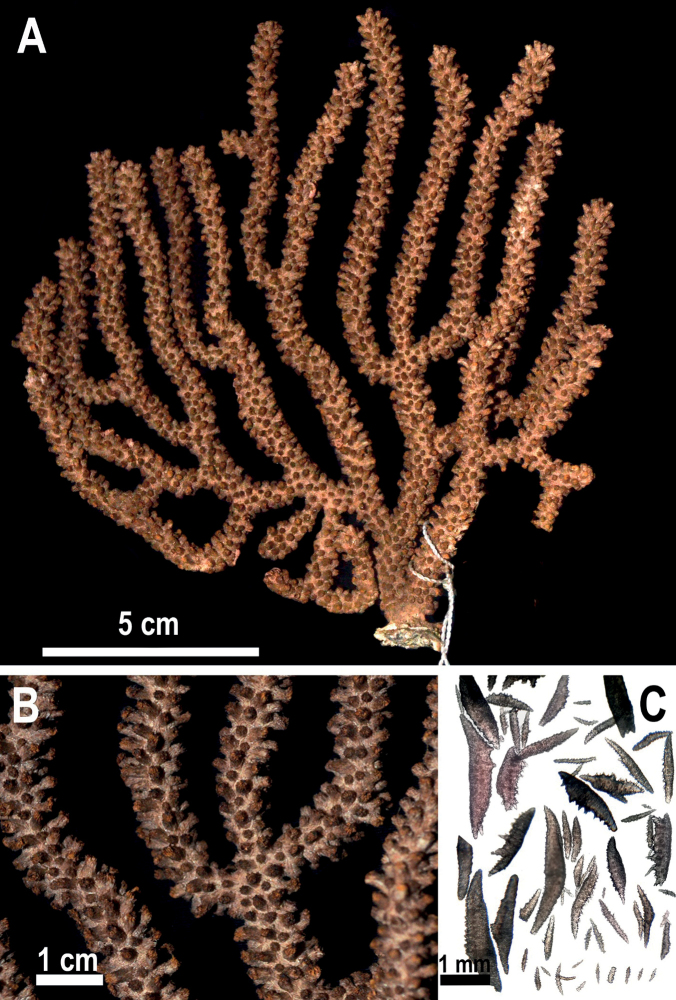
*Muriceamolinai* sp. nov. **A** holotype USNM 1606637 **B** holotype detail of branches and calyces **C** anthocodial and coenenchymal sclerites.

*Muriceamolinai* sp. nov. colony coloration is gray with dark gray calyces and a creamy light gray coenenchyme (Fig. 6A, B). The colony is flabellate growing in one plane and laterally branched, reaching 15 cm in height and 14.7 cm in width (Table 1). The holdfast is 22 mm long and 13 mm wide. Two main stems arise from the holdfast one with 2.4 cm and the second one with 4.5 cm in height, subdividing laterally in stems of 7 mm in diameter (Fig. 6A). The growth branching pattern is upward, except six secondary branches, on both sides of the colony, which display more of a downward-growing trend. Terminal branches are up to 8.7 cm tall with blunt ends of 5 mm diameter. Calyces are tubular, 3 mm in height with a 1.5 mm diameter, with an imbricated arrangement throughout the colony (Fig. 6A, B).

###### Holotype sclerites.

**Figure 7.**
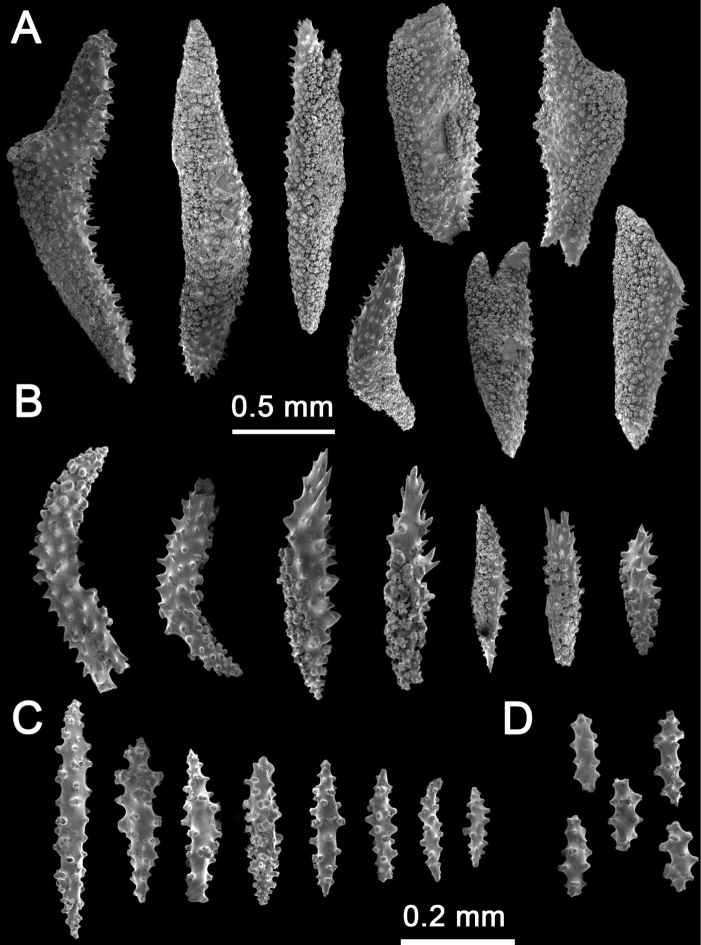
*Muriceamolinai* sp. nov. SEM**A, B** calycular and coenenchymal sclerites **C** axial sclerites **D** anthocodial sclerites.

The sclerites of the outer coenenchyme and calyx are unilateral (weakly) spinous spindles and tuberculated spindles (1.2–2.5 mm in length) (Figs 6C, 7A–C). The spindles are curved or straight; most of them have acute ends or are bifurcated; tuberculated spindles with blunt ends are rare. The inner coenenchymal sclerites are mostly straight or curved spindles with acute ends (0.2–1.2 mm length), but there are rare blunt spindles. Anthocodial sclerites are small tuberculated spindles (1.1–1.8 mm length) with acute ends and rods with marginal lobes and acute ends (Fig. 7D). The color of the outer coenenchymal sclerites is gray, or gray with pale amber areas in the largest ones. The inner coenenchymal and anthocodial sclerites are colorless (Fig. 6C).

###### Morphological variation.

**Figure 8.**
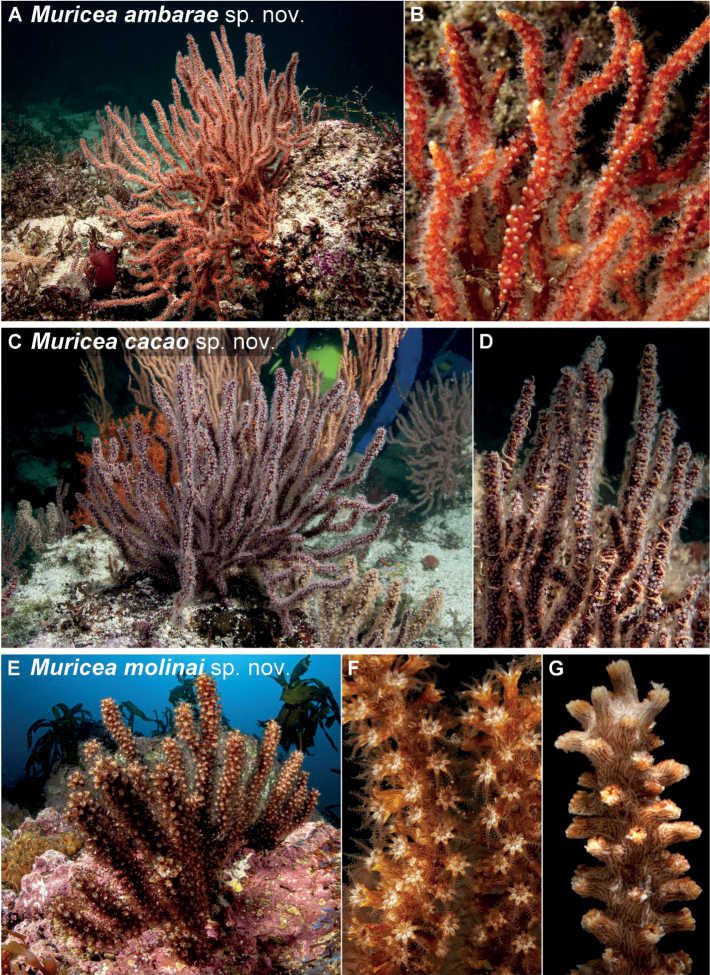
Sea fan colonies photographed *in situ***A, B***Muriceaambarae* sp. nov. **C, D***Muriceacacao* sp. nov. **E–G***Muriceamolinai* sp. nov. Photographs by Carlos Sánchez.

All twelve *Muriceamolinai* sp. nov. colony specimens collected, including the paratypes ranging between 7 and 21 cm height (Fig. 8E–G; Suppl. material 1: fig. S5A, B), were morphologically similar to the holotype. No detectable color variability or different colony growth pattern was observed among the examined colonies of *M.molinai* sp. nov. (Table 1). The length of the tubular calyces is the main morphological variability among *M.molinai* sp. nov. specimens, where several colonies have longer calyces (> 4 mm in length throughout the colony) than other colonies with shorter calyces (< 2 mm in length). The polyps of live colonies have white neck with translucent brown tentacles (Fig. 8F; Suppl. material 1: fig. S6C).

###### Habitat and distribution.

*Muriceamolinai* sp. nov. is absent in the Gulf of California but overlaps its distribution and habitat with *Muriceaambarae* sp. nov. and *Muriceacacao* sp. nov. along the southwest coast of the Baja California Peninsula (Fig. 1B–D). *Muriceamolinai* sp. nov. was collected between Bahía Magdalena and Cabo San Lucas; but it was absent at the Punta Abreojos site (Fig. 1D).

###### Remarks.

Live colonies of *M.molinai* sp. nov. (Fig. 8E-G) and *Muriceasquarrosa* (Breedy and Guzman 2015) (Suppl. material 1: fig. S6A–M) are similar, but the shape of the calyces and the differences in the diameter of the branches are the most evident diagnostic feature to distinguish them. *Muriceamolinai* sp. nov. has a distinctive colony coloration, being light gray in the coenenchyme, and dark gray almost black in the calyces which extend sparsely from the base to the tip of the branches (Fig. 8E–G; Suppl. material 1: figs S5A, B, S6C–F). Preserved colonies of *Muriceasquarrosa* are all reddish-brown and without calyces on the base and main stem. The long unilateral spinous spindles (2.5 mm) of *M.molinai* sp. nov. are one of the longest known spinous spindles among the species of the genus *Muricea* and without clubs in the coenenchyme and calyx while the spindles of *M.squarrosa* are smaller, only up to 1.3 mm in length (Verrill 1868 reported spindles of 1.8 mm), and has club-like spindles in the calyx (absent in *M.molinai* sp. nov.). The main differences to separate *Muriceaacervata*, *M.hispida*, *M.tubigera* and *M.molinai* sp. nov. (species belonging to the *M.squarrosa* species-group) are the size and arrangement of the calyces and the size of the sclerites. *Muriceatubigera* has the largest calyces (5 mm length) while *M.molinai* sp. nov. have calyces of 3 mm length. It is proposed that *Muriceamolinai* sp. nov. be included in the *Muriceasquarrosa* species-group previously erected by Breedy and Guzman (2015).

###### Etymology.

*Muriceamolinai* sp. nov. is named in memory of Dr. José Mario Molina Pasquel y Henríquez (1943–2020), the first Mexican in 1995 to win the Nobel Prize in Chemistry. Molina played a vital role in the discovery of the Antarctic ozone hole demonstrating that chlorofluorocarbon gases were the cause of the deterioration of the ozone layer.

### ﻿Molecular analysis

For several sea fan genera, the mtMutS gene is conserved to species level; but it is divergent enough to discriminate among genera and species-groups in octocorals. The partial fragment used for the phylogenetic reconstruction was highly conserved in *Muricea*, nonetheless and even with a lack of strong node support, *Muriceaambarae* sp. nov. and *Muriceacacao* sp. nov. were separated in a well-defined clade together with the nominal species *Muriceafruticosa*. In the case of *Muriceamolinai* sp. nov., although not falling into a clade and showing unresolved relationships, it is clearly distinct from these last two new species and close to the nominal species *Muriceasquarrosa* and *Muriceahebes*. The comparison of these mtMutS sequences with *M.plantaginea*, *M.californica* and *M.squarrosa* support the morphological evidence that, *M.ambarae* sp. nov., *M.cacao* sp. nov. and *M.molinai* sp. nov. are new species (Fig. 10; Suppl. material 1: table S1).

## ﻿Discussion

**Figure 9.**
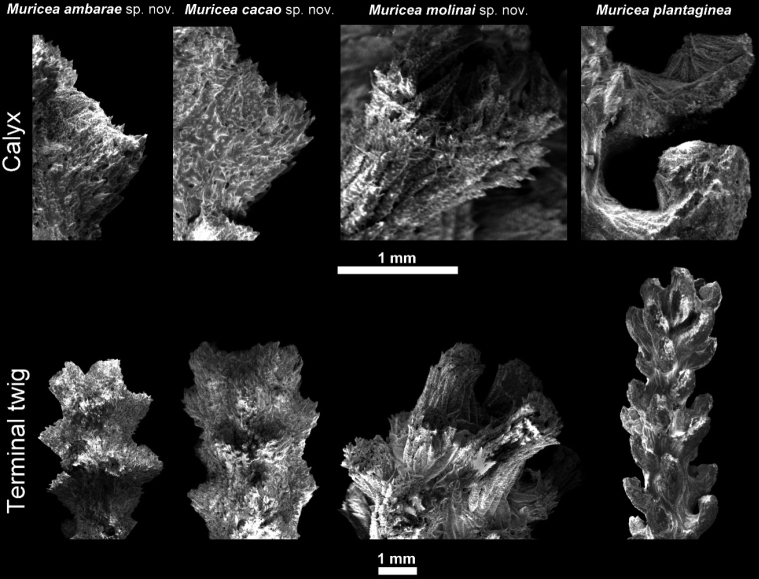
Comparison of the SEM images of calix (upper row) and terminal twig (bottom row) of sea fans *Muriceaambarae* sp. nov., *M.cacao* sp. nov., *M.molinai* sp. nov. and the nominal species *M.plantaginea*.

Sea fan species assemblages have a clear latitudinal regionalization in the Gulf of California (Ulate et al. 2016). The southern region has environmental conditions influenced by the tropical surface water mass typical of the Mexican Province (Brusca and Wallerstein 1979; Hasting 2000; Portela et al. 2016). This southern region has the highest species richness and density of sea fan species of the genera *Pacifigorgia* Bayer, 1851 and *Leptogorgia* Milne Edwards, 1857 (Ulate et al. 2016). The Midriff Archipelago Region (MAR) (Fig. 1A) is the coldest region in the Gulf of California due to intense tidal currents and topographic upwelling (Lavín and Marinone 2003; López et al. 2006; Ulate et al. 2016), where sea fan species richness and abundance are numerically dominated by *M.austera*, *M.fruticosa* and *M.plantaginea*. *Muriceaambarae* sp. nov. and *M.cacao* sp. nov. share their habitat (5–20 m depth) in this region (Fig. 1B, C), where *M.plantaginea* is the most abundant species. We show SEM morphological evidence that the three new species have a distinct calyx and terminal twig morphology that differs from *M.plantaginea* (Fig. 9). *Muriceaambarae* sp. nov. and *M.cacao* sp. nov. were first reported in the MAR as undescribed species labeled as: *Muricea* sp. 2 and *Muricea* sp. 5 in Ulate et al. (2016). *Muriceaaustera*, *M.fruticosa* and *M.plantaginea* are also numerically dominant at > 40 m seafloor depth associated with low temperatures in the southwestern region of the Gulf of California between Loreto and Cabo San Lucas. However, *M.ambarae* sp. nov. and *M.cacao* sp. nov. have not been observed in the southern region of the Gulf of California (Ulate et al. 2016); they are also distributed along the Pacific coast of the Baja California Peninsula, Mexico, between Cabo San Lucas and Punta Abreojos, Baja California Sur, Mexico (Fig. 1B, C). *Muriceamolinai* sp. nov. is distributed in shallow waters (< 20 m) in the Bahía Magdalena region, but it can be observed deeper >50 m in Cabo San Lucas (Fig. 1D). Bahía Magdalena is located at a transitional biogeographic region between the Mexican Province and the California Current Province with seasonal cold waters (Hasting 2000), which could explain the preference of *Muricea* species for temperate habitats.

**Figure 10.**
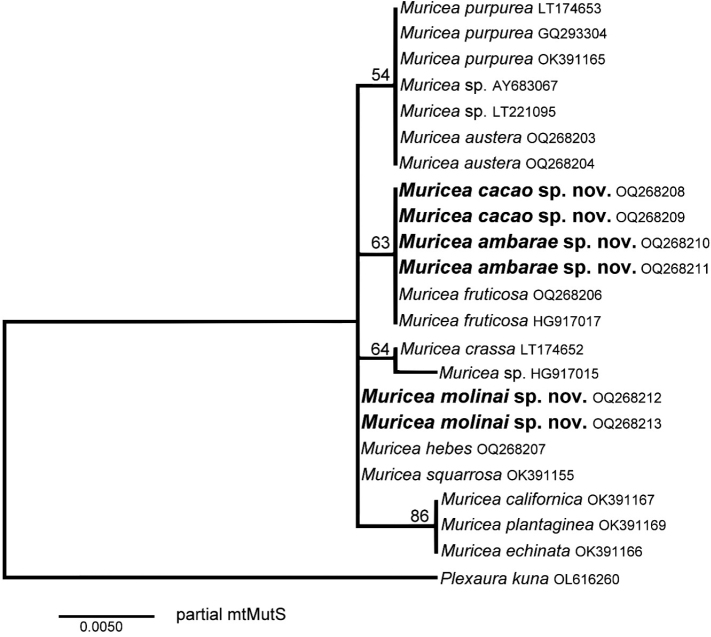
Maximum-likelihood phylogenetic reconstruction of partial mtMutS gene for *Muricea* reported for the Pacific Ocean showing in bold the sequences of the present study of *Muriceaambarae* sp. nov., *Muriceacacao* sp. nov. and *Muriceamolinai* sp. nov. Numbers above the branches are bootstrap percentage values.

The molecular analysis of species of the genus *Muricea* and other sea fan genera distributed in the Eastern Pacific is still incipient (Herrera et al. 2010; Gómez 2012; Vargas et al. 2014; Poliseno et al. 2017). Mitochondrial genetic sequences deposited in GenBank, National Center for Biotechnology (NCBI) are available for only seven out the 21 *Muricea* nominal species distributed in the Eastern Pacific: *Muriceacalifornica*, *Muriceacrassa* Verrill, 1869, *Muriceaechinata*, *Muriceafruticosa*, *Muriceaplantaginea*, *Muriceapurpurea* and *Muriceasquarrosa* (Herrera et al. 2010; Poliseno et al. 2017) and several other *Muricea* spp. (Wirshing et al. 2005; Vargas et al. 2014; Ament-Velásquez et al. 2016) (Suppl. material 1: table S1). Few studies have tried so far to infer the phylogenetic relationships of species of the genera *Muricea* with single mitochondrial genes or complete mitogenomes. Therefore, there is still no comprehensive knowledge for taxonomic species delimitation for most *Muricea* species distributed in the Eastern Pacific (Vargas et al. 2014; Poliseno et al. 2017). This lack of morphological and genetic knowledge is mainly the reason that *Muricea* has historical taxonomic problems in species identification of specimens observed in the field and from preserved material (Breedy and Guzman 2015; 2016a, b; Ulate et al. 2016) as well as the lack of systematic benthic surveys to cover most of the rocky and pebbled sea floor habitats in the northwest Mexico region (Hernández et al. 2021). Despite these historical limitations, our molecular evidence (mtMutS gene) supports the conclusion that the three species discovered in the present study are indeed new species compared with other nominal species distributed in the region of study (Figs 1, 10).

Breedy and Guzman (2015) redescribed the genus *Eumuricea* Verrill, 1869 in the Eastern Pacific and reclassified it within *Muricea*, proposing that it be included in the *Muriceasquarrosa* species-group, which includes species with tubular calyces. We propose to include *Muriceamolinai* sp. nov. in the *M.squarrosa* species-group suggesting that *M.molinai* sp. nov. and *M.squarrosa* have anti-tropical distribution inhabiting subtropical latitudes. Anti-tropical species are populations with a disjunct distribution, that is, the ancestor originated on one side of the tropics, dispersing later and occupying geographical areas in the opposite hemisphere, being absent within the rocky Mexican Province and Central American gap, as has been observed in reef fish species assemblages (Hasting 2000). Later, Breedy and Guzman (2016a) redescribed several *Muricea* species, proposing the *Muriceaaustera* species-group, the *M.fruticosa* species-group (where we included *Muriceaambarae* sp. nov. and *Muriceacacao* sp. nov.), and the *M.plantaginea* species-group as mentioned earlier. The practical purposes of these *Muricea* species-groups (which do not necessarily have a phylogenetic relationship) and the precise morphological descriptions of these nominal species have facilitated species identification in field ecological studies, allowing the description of new species such as the most recently described species *Muriceasubtilis* Breedy & Guzman, 2016 (Breedy and Guzman 2016b). Although the dominant octocoral genera in the Eastern Tropical Pacific are *Leptogorgia*, *Muricea*, and *Pacifigorgia* (Breedy and Cortés 2015; Abad et al. 2022), it is still difficult to infer the gamma species richness in the Northern Mexican Pacific because several new species still await formal description. Three species of *Leptogorgia* were recently discovered in the Gulf of California (Hernández et al. 2021). The discovery of these three frequent and abundant new *Muricea* species reported in the present study is due to the previous lack of research effort carried out in both the Midriff Archipelago Region and the Bahía Magdalena region (Fig. 1A), and overall, the historical lack of taxonomic knowledge of *Muricea* species in the northwest region of Mexico.

## Supplementary Material

XML Treatment for Muricea ambarae

XML Treatment for Muricea cacao

XML Treatment for Muricea molinai

## ﻿Additional information

### Author ORCIDs

Osvaldo Hernández https://orcid.org/0000-0001-6195-149X

Jaime Gómez-Gutiérrez https://orcid.org/0000-0003-2516-897X

Carolina Galván-Tirado https://orcid.org/0000-0003-3973-256X

Carlos Sánchez https://orcid.org/0000-0001-7199-1268

### Data availability

All of the data that support the findings of this study are available in the main text or Supplementary Information.

## Supplementary materials

Supplementary material 1 Supplementary data Data type morphological, images, molecular data Explanation note We showed the paratypes images of the three sea fans described species. Also, the *in situ* images comparison between the described species with other nominal species of *Muricea*. The methodology of the extraction and molecular analysis is included, and the table of accession numbers in GenBank. https://binary.pensoft.net/file/880619This dataset is made available under the Open Database License (http://opendatacommons.org/licenses/odbl/1.0/). The Open Database License (ODbL) is a license agreement intended to allow users to freely share, modify, and use this Dataset while maintaining this same freedom for others, provided that the original source and author(s) are credited.Osvaldo Hernández, Jaime Gómez-Gutiérrez, Carolina Galván-Tirado, Carlos Sánchez
