# Supplementary material for: ﻿Three new species of the sea fan genus Muricea (Cnidaria, Octocorallia, Plexauridae) from the northwest region of Mexico
Source: Zookeys. 2023 Jul 18;1169:333–52. doi: 10.3897/zookeys.1169.89651 (PMC10369450; doi:10.3897/zookeys.1169.89651)
Supplement: Supplementary material 1 — Supplementary data [file zookeys-1169-333_article-89651__-s001.docx]

**Supplementary Material**

**Three new species of the sea fan genus *Muricea* (Cnidaria, Octocorallia, Plexauridae) from the northwest region of Mexico**

Osvaldo Hernández ^1,3^, Jaime Gómez-Gutiérrez^1^, Carolina Galván-Tirado^2,3^, Carlos Sánchez^3^

**1** *Departamento de Plancton y Ecología Marina, Centro Interdisciplinario de Ciencias Marinas, Instituto Politécnico Nacional, Av. IPN, s/n, CP 23096, La Paz, Baja California Sur, Mexico* **2** *Consejo Nacional de Ciencia y Tecnología, Av. Insurgentes Sur 1582, Col. Crédito Constructor, Alcaldía Benito Juárez, C.P. 03940, Ciudad de Mexico, Mexico* **3** *Departamento de Ciencias Marinas y Costeras, Universidad Autónoma de Baja California Sur, Carretera al sur km 5.5, CP 23080, La Paz, Baja California Sur, Mexico*

Corresponding author: *Carlos Sánchez* ([csanchez@uabcs.mx](mailto:csanchez@uabcs.mx))

**Running head:** Three new species of *Muricea* from the Mexican Pacific

**
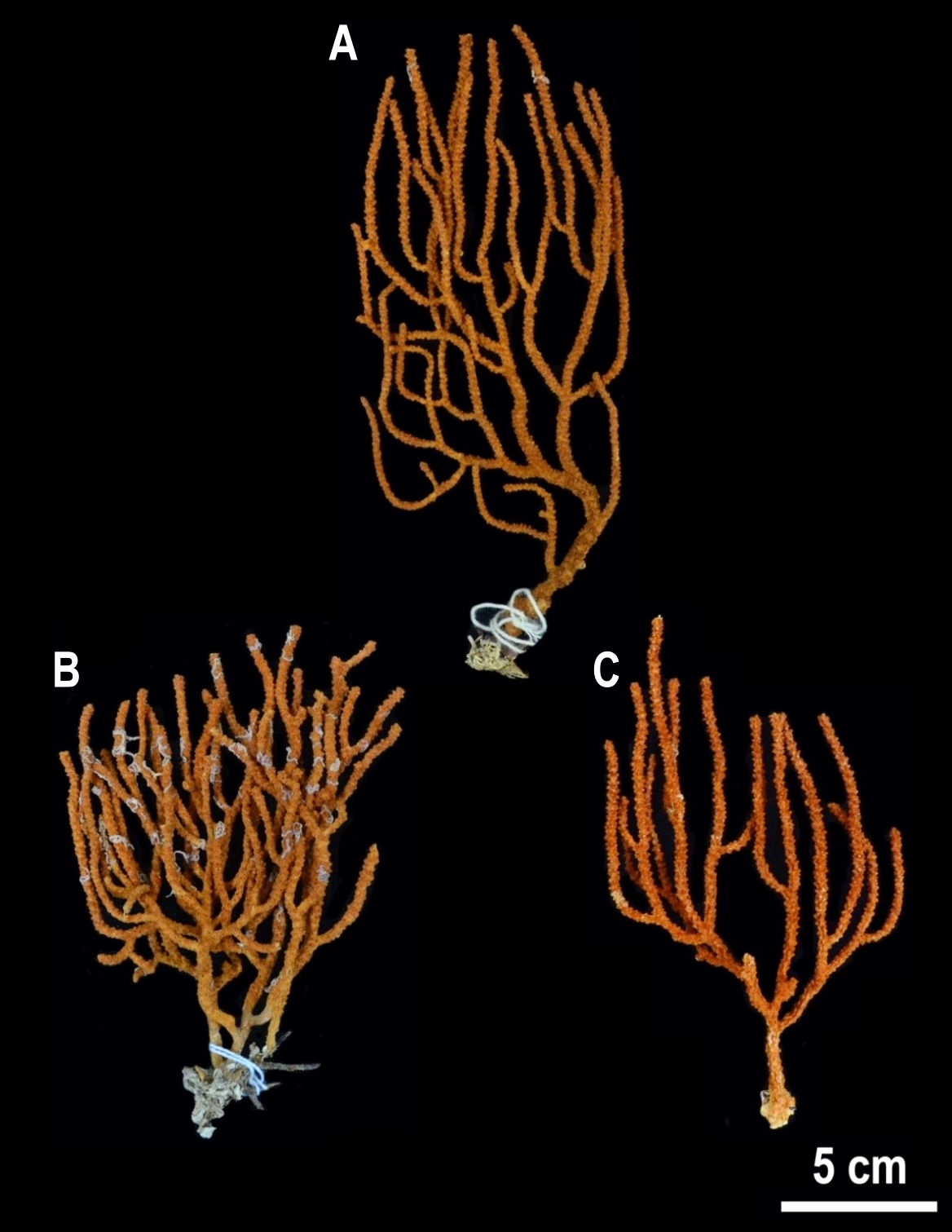
**

**Figure S1.** *Muricea ambarae* sp. nov. paratypes collected in the northwest region of Mexico to show intraspecific colony morphology variability **A** USNM 1606630: San Esteban Island (Punta Noroeste), Sonora, Mexico **B** USNM 1606631: Bahía de Las Ánimas (Los Choros), Baja California, Mexico **C** USNM 1606632: Punta Abreojos, Pacific coast of Baja California Sur, Mexico.

**
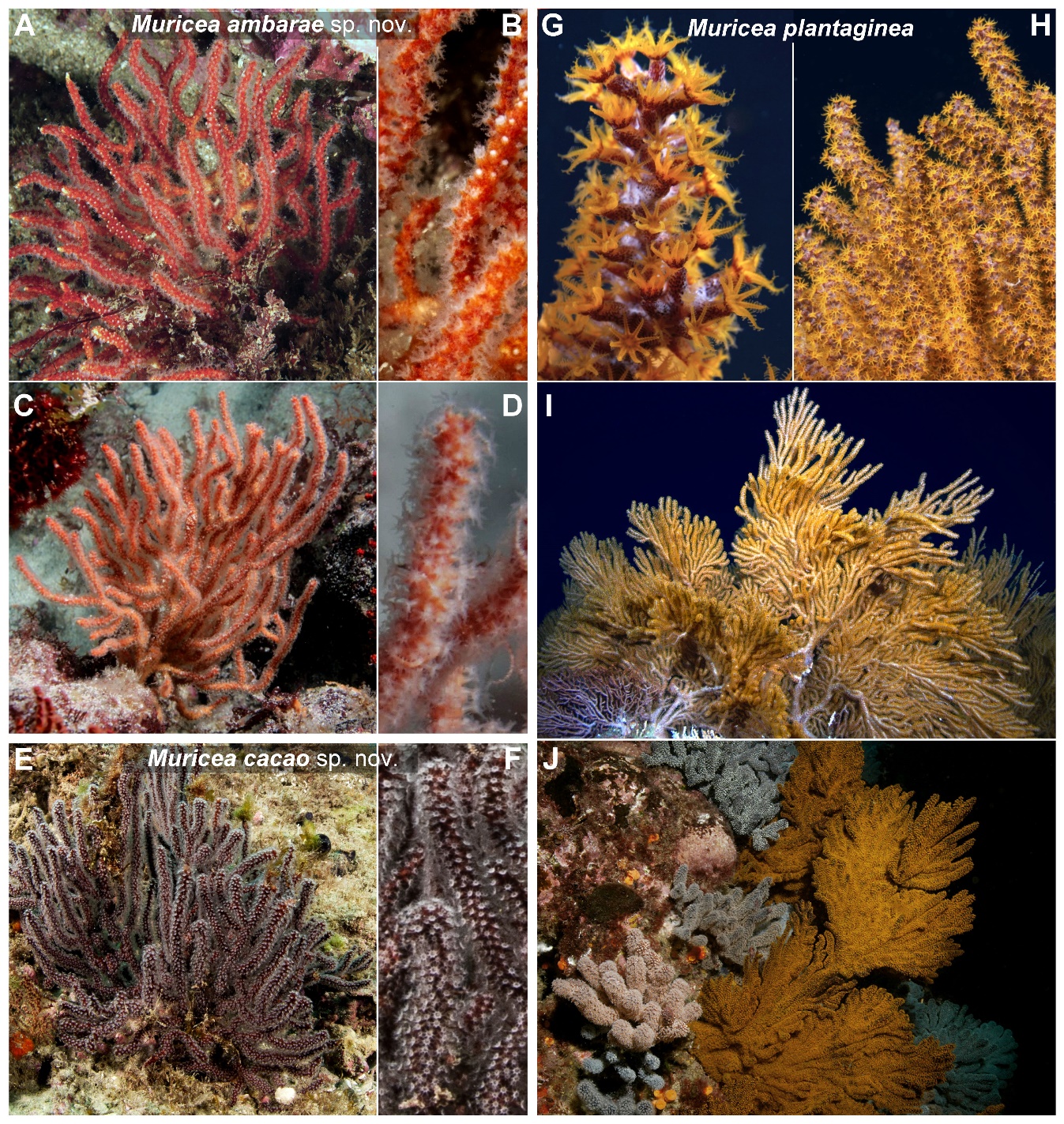
Figure S2.** Comparison of the sea fans colonies *in situ*, underwater images of **A-D** *Muricea ambarae* sp. nov. **E-F** *Muricea cacao* sp. nov. **G-I** *Muricea plantaginea*. Photographs **A-I** Carlos Sánchez **J** Image from Breedy and Guzman (2016).


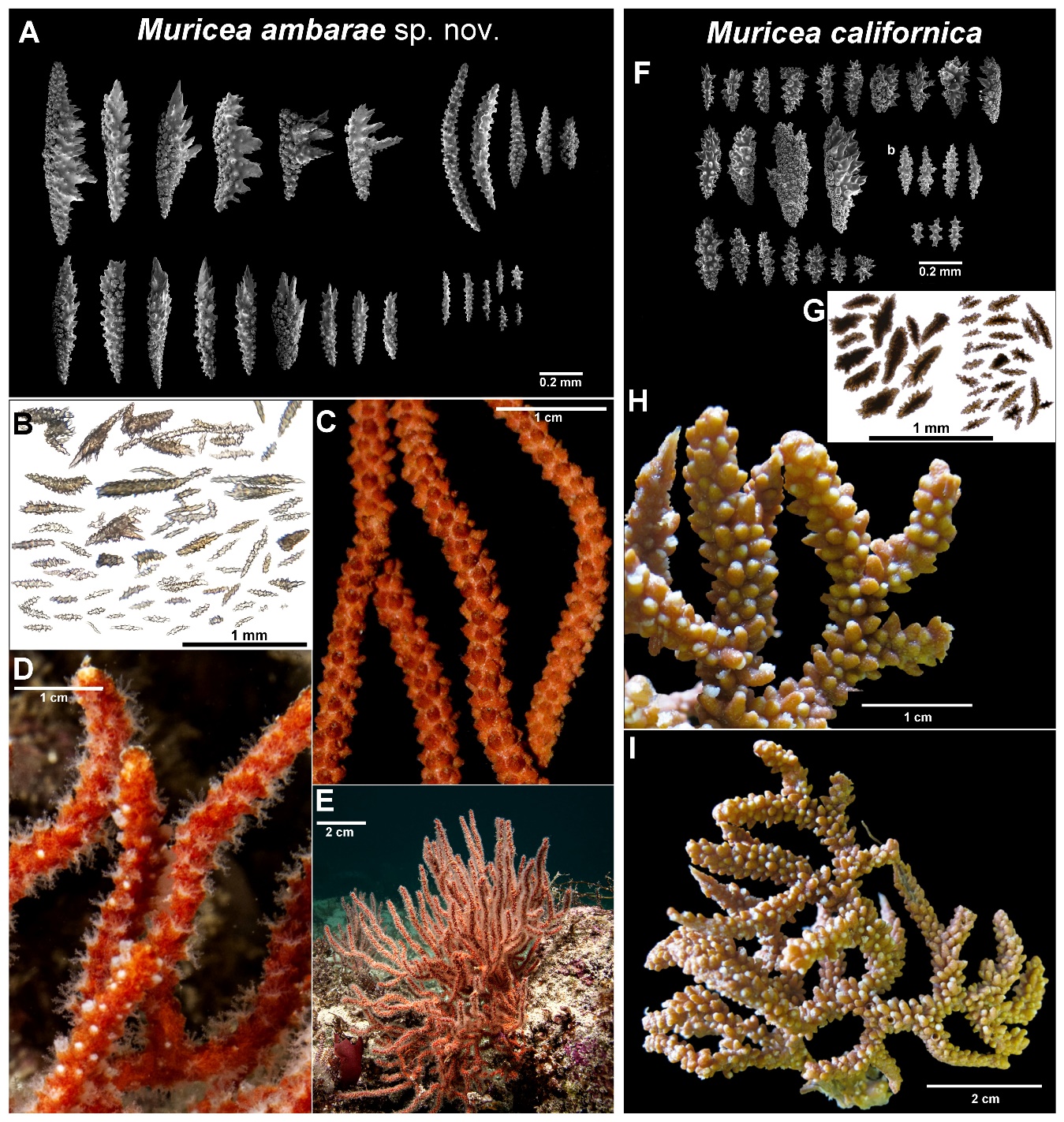
**Figure S3.** Comparison of the sea fans colonies, sclerites types, dry material branches and colony and *in situ*, underwater images of **A-E** *Muricea ambarae* sp. nov. with **F-I** *Muricea californica*. Photographs **A-C** present study **D-E** by Carlos Sánchez **F-I** Images modified from Breedy and Guzman (2016).

| 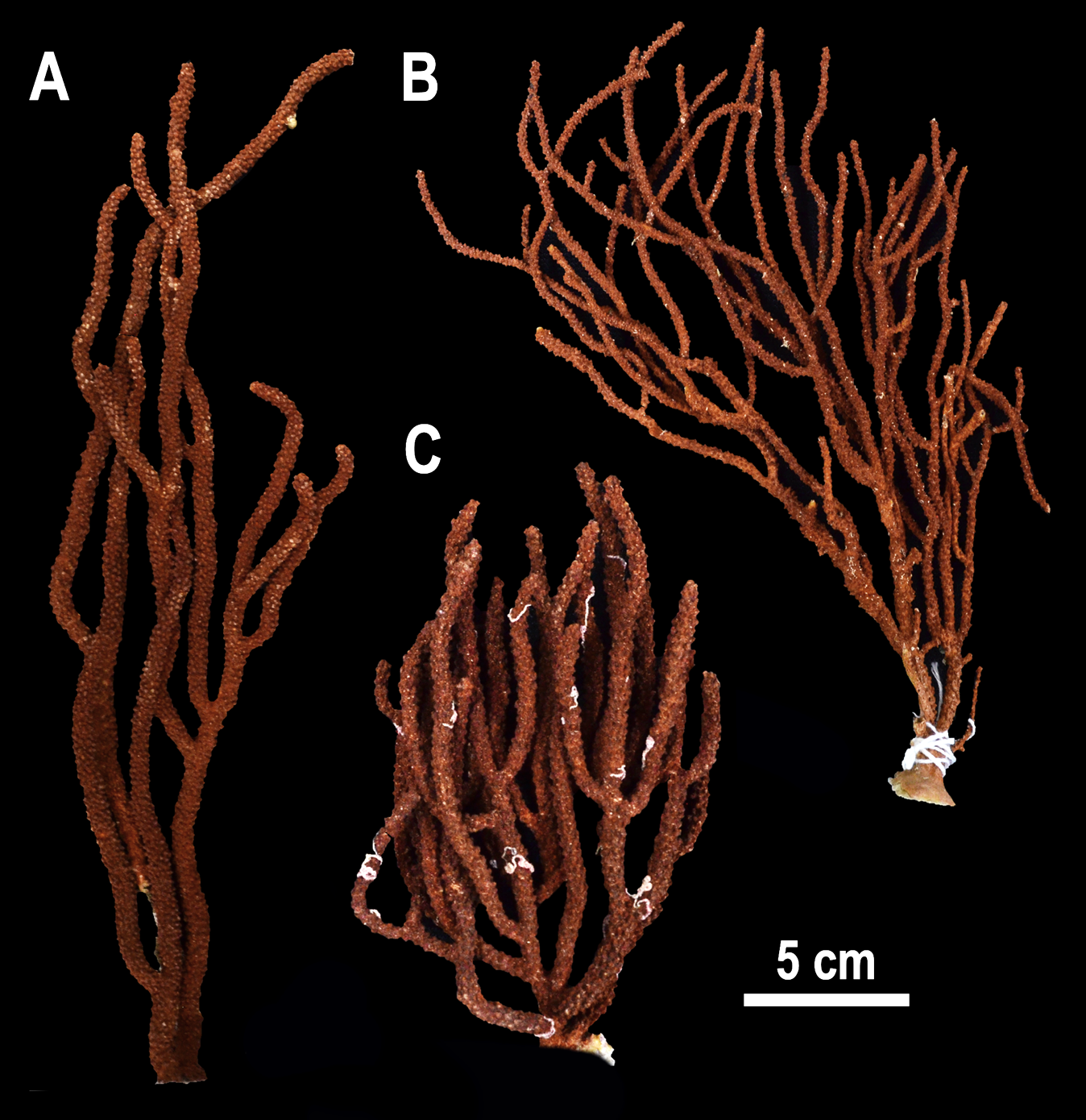 |
| --- |

**Figure S4.** *Muricea cacao* sp. nov. paratypes collected in the northwest region of Mexico to show intraspecific colony morphology variability **A** USNM 1606635: Salsipuedes Island (Caleta Falsa Norte), Baja California, Mexico **B** USNM 1606636: Bahía de los Ángeles (Punta Pescador), Baja California, Mexico **C** USNM 1606634: San Marcos Island (El Faro-Lobera), Baja California Sur, Mexico.


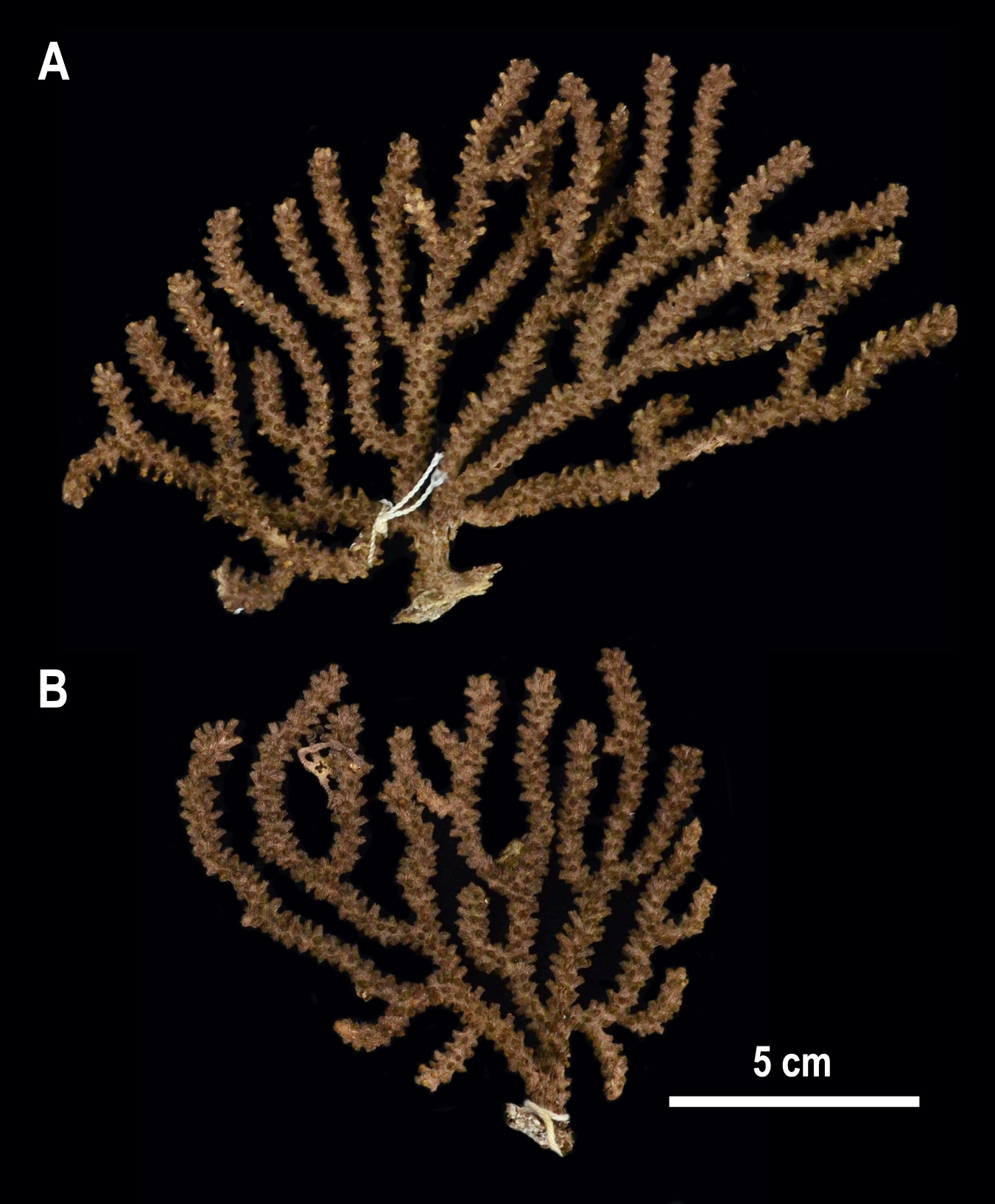
**Figure S5.** *Muricea molinai* sp. nov. paratypes collected in the northwest region of Mexico to show intraspecific colony morphology variability **A** USNM 1606638: Todos Santos, Punta Lobos (Bajo Fondo del Medio), Pacific coast of Baja California Sur, Mexico **B** USNM 1606639: Bahía Santa María, Cabo San Lázaro (Roca del Cabito), Pacific coast of Baja California Sur, Mexico.


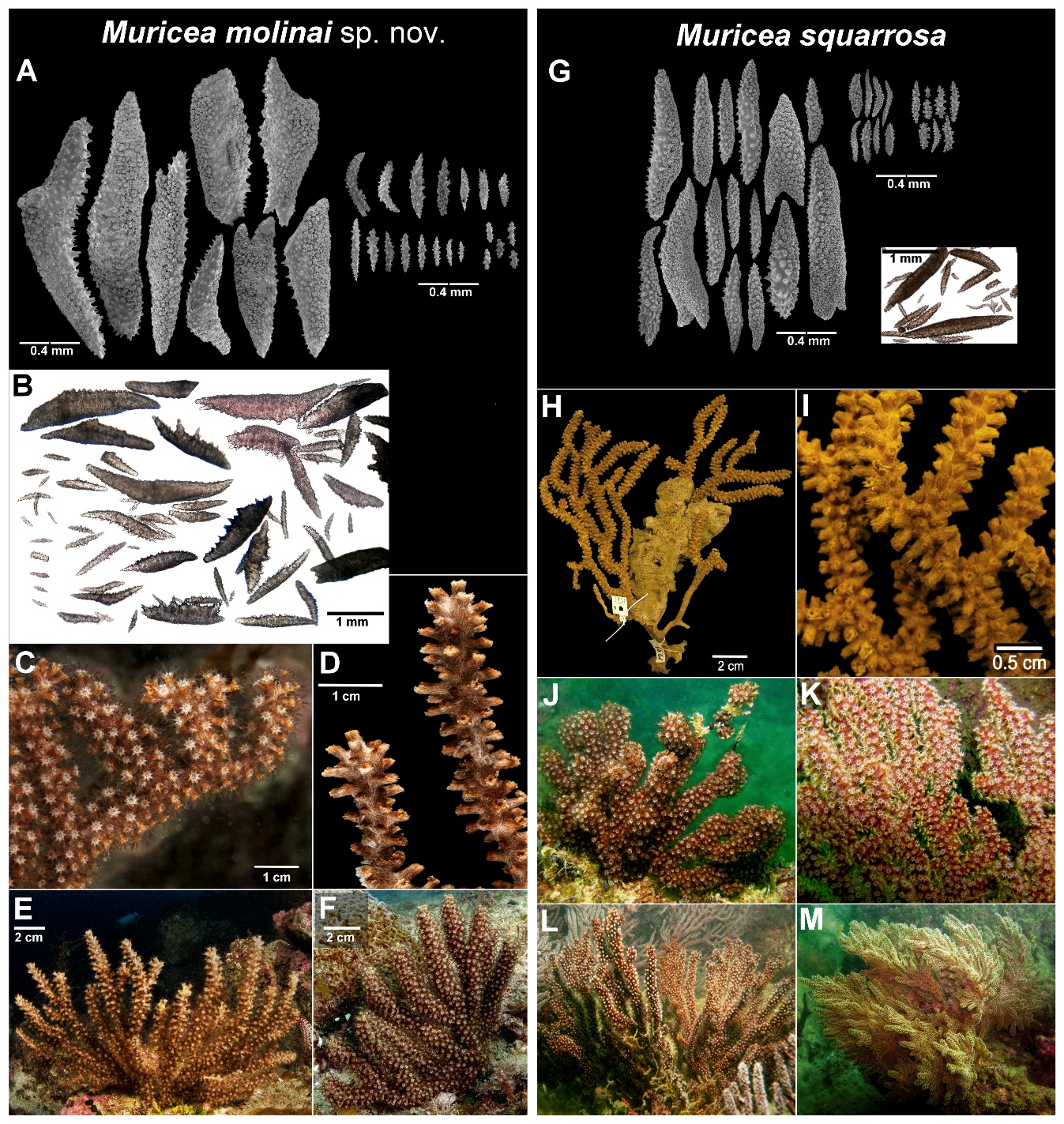
**Figure S6.** Comparison of the sea fans colonies, sclerites types, dry material branches and colony and in situ, underwater images of **A-F** *Muricea molinai* sp. nov. with **G-M** *Muricea squarrosa*. Photographs **A-B** present study **C-F** by Carlos Sánchez **G-M** Images modified from Breedy and Guzman (2015).

**Data S1**. Details of the molecular analyses.

The genomic DNA was extracted from ten sea fan colonies collected in the Mexican Pacific using the Qiagen DNeasy Blood & Tissue kit (Qiagen, Valencia, CA) following the manufacturer’s protocol. A pair of primers were designed to amplify a fragment of ~931 bp from the mitochondrial MutS gene (mtMutS): MutS 1-F CCGAATCTGCTATTGGTTTAG and MutS 1-R AATTTTTGTTAAACCACTCTG with Primer 3Web (http://primer3.ut.ee; Untergasser et al. 2012). The reactions of the polymerase chain reaction (PCR) were carried out in 30 µl total volume containing final concentrations of 50-100 ng/µl DNA template, 0.3 mM dNTPs, 2 mM MgCl_2_, 0.8 mg/ml Bovine Serum Albumin (BSA), 1 U Taq DNA polymerase (Invitrogen, Inc., Carlsbad, CA), 0.3 µM each primer, and 1X of reaction buffer. The PCR profile was carried out as follows: one cycle of 95 °C for 5 min, 35 cycles of 95 °C for 1 min, 50 °C for 1 min, 72 °C for 1 min and a final extension of 72 °C for 10 min. Each PCR product was sequenced by Macrogen Co. in both DNA strands. Sequence edition, strands assembling, and alignments were performed in Geneious Prime software 2022.2 (Kearse et al. 2012). The Tamura 3-parameter substitution model was used as the best-fit model for the dataset by jModeltest 2.1.6 for the tree reconstruction (Darriba et al. 2012). The phylogenetic tree was performed with maximum likelihood (ML) using Mega7 (Kumar et al. 2016) with 1000 bootstrap replicates. *Plexaura kuna* (OL616260 Genbank accession) (Muthye et al. 2022), a sister taxon of *Muricea,* was used as outgroup. Thus, a total of 23 mtMutS sequences were analyzed in the phylogenetic tree (Fig. 10).

**Table S1.** *Muricea* species included in the molecular analyses, and their corresponding internal catalog number and GenBank accession numbers. Data from the outgroup is included.

| **Species** | **Catalog number** | **Sampling location** | **Genbank ID** | **Reference** |
| --- | --- | --- | --- | --- |
| *Muricea austera* Verrill, 1869 | IM10_21 | Islote, San Juanico Island, Islas Marías Archipelago, Nayarit, Mexico | OQ268203 | Present study |
| *Muricea austera* Verrill, 1869 | IM10_17 | Islote, San Juanico Island, Islas Marías Archipelago, Nayarit, Mexico | OQ268204 | Present study |
| *Muricea californica* Aurivillius, 1931 | ECU005 | NA | OK391167 | Vergara-Florez, unpublished |
| *Muricea crassa* Verrill, 1869 | NA | Coiba National Park, Panama | LT174652 | Poliseno et al. (2017) |
| *Muricea echinata* Verrill, 1866 | ECU083 | NA | OK391166 | Vergara-Florez, unpublished |
| *Muricea fruticosa* Verrill, 1869 | TV_11 | Bahía Magdalena, Baja California Sur, Mexico | OQ268206 | Present study |
| *Muricea fruticosa* Verrill, 1869 | HMG94 | Coiba Island, Panama | HG917017 | Vargas et al. (2014) |
| *Muricea hebes* Verrill, 1864 | UABCS-PFA-CN-276 | Rio de La Plata shipwreck, Acapulco, Guerrero, Mexico | OQ268207 | Present study |
| *Muricea plantaginea* (Valenciennes, 1846) | ECU004 | NA | OK391169 | Vergara-Florez, unpublished |
| *Muricea purpurea* Verrill, 1868 | NA | Coiba National Park, Panama | LT174653 | Poliseno et al. (2017) |
| *Muricea purpurea* Verrill, 1868 | USNM 1016584 | Panama Bay, Panama | GQ293304 | Herrera et al. (2010) |
| *Muricea purpurea* Verrill, 1868 | ECU021 | NA | OK391165 | Vergara-Florez, unpublished |
| *Muricea squarrosa* Verrill, 1869 | ECU044 | NA | OK391155 | Vergara-Florez, unpublished |
| *Muricea* sp. | CAS-1007376 | Rocas Alijos, Mexico | AY683067 | Wirshing et al. (2005) |
| *Muricea* sp. | HMG81 | Coiba Island, Panama | HG917015 | Vargas et al. (2014) |
| *Muricea* sp. | MZUCR-OCT 0023 | Costa Rica | LT221095 | Ament-Velásquez et al. (2016) |
| *Muricea cacao* **sp. nov.** holotype | USNM 1606633 | San Marcos Island (El Faro-Lobera), Baja California Sur, Mexico | OQ268208 | Present study |
| *Muricea cacao* **sp. nov.** paratype | USNM 1606635 | Salsipuedes Island (Caleta Falsa Norte), Baja California, Mexico | OQ268209 | Present study |
| *Muricea ambarae* **sp. nov.** paratype | USNM 1606630 | San Esteban Island (Punta Noroeste), Sonora, Mexico | OQ268210 | Present study |
| *Muricea ambarae* **sp. nov.** holotype | USNM 1606629 | San Esteban Island (Punta Sureste), Sonora, Mexico | OQ268211 | Present study |
| *Muricea molinai* **sp. nov.** paratype | USNM 1606639 | Bahía Santa María, Cabo San Lázaro (Roca del Cabito), Baja California Sur, Mexico | OQ268212 | Present study |
| *Muricea molinai* **sp. nov.** | UABCS-PFA-CN-164 | Bajo El Golfito, Cabo San Lucas, Baja California Sur, Mexico | OQ268213 | Present study |
| *Plexaura kuna* Lasker, Kim, Coffroth, 1996 (outgroup) | NA | Bocas del Toro, Panama | OL616260 | Muthye et al. (2022) |

**References of Supplementary Material**

Ament-Velásquez SL, Breedy O, Cortés J, Guzman HM, Wörheide G, Vargas S (2016) Homoplasious colony morphology and mito-nuclear phylogenetic discordance among Eastern Pacific octocorals. Molecular Phylogenetics and Evolution 98: 373–381. <https://doi.org/10.1016/j.ympev.2016.02.023>

Breedy O, Guzman HM (2015) A revision of the genus *Muricea* Lamouroux, 1821 (Anthozoa, Octocorallia) in the eastern Pacific. Part I: *Eumuricea* Verrill, 1869 revisited. Zookeys 537: 1–32. <https://doi.org/10.3897/zookeys.537.6025>

Breedy O, Guzman HM (2016) A revision of the genus *Muricea* Lamouroux, 1821 (Anthozoa, Octocorallia) in the eastern Pacific. Part II. ZooKeys 581: 1–69. <https://doi.org/10.3897/zookeys.581.7910>

Darriba D, Taboada GL, Doallo R, Posada D (2012) jModelTest 2: more models, new heuristics and parallel computing. Nature Methods 9: 772. <https://doi.org/10.1038/nmeth.2109>

Herrera S, Baco A, Sánchez JA (2010) Molecular systematics of the bubblegum coral genera (Paragorgiidae, Octocorallia) and description of a new deep-sea species. Molecular Phylogenetics and Evolution 55:123–135. <https://doi.org/10.1016/j.ympev.2009.12.007>

Kearse M, Moir R, Wilson A, Stones-Havas S, Cheung M, Sturrock S, Buxton S, Cooper A, Markowitz S, Duran C, Thierer T, Ashton B, Meintjes P, Drummond A (2012) Geneious Basic: an integrated and extendable desktop software platform for the organization and analysis of sequence data. Bioinformatics 28:1647–1649 <https://doi.org/10.1093/bioinformatics/bts199>

Kumar S, Stecher G, Tamura K (2016) MEGA7: Molecular Evolutionary Genetics Analysis Version 7.0 for Bigger Datasets. Molecular Biology and Evolution 33: 1870–1874. <https://doi.org/10.1093/molbev/msw054>

Muthye V, Mackereth CD, Stewart JB, and Lavrov D.V (2022). Large dataset of octocoral mitochondrial genomes provides new insights into mt-mutS evolution and function. DNA Repair 110:103273. <https://doi.org/10.1016/j.dnarep.2022.103273>

Poliseno A, Breedy O, Eitel M, Wöerheide G, Guzman HM, Krebs S, Blum H, Vargas S. (2017) Complete mitochondrial genome of *Muricea crassa* and *Muricea purpurea* (Anthozoa: Octocorallia) from the eastern tropical Pacific. bioRXiv. <https://doi.org/10.1101/042945>

Untergasser A, Cutcutache I, Koressaar T, Ye J, Faircloth BC, Remm M, Rozen SG (2012) Primer3—new capabilities and interfaces. Nucleic Acids Research, 40(15): e115. <https://doi.org/10.1093/nar/gks596>

Vargas S, Guzman HM, Breedy O, Wörheide G (2014) Molecular phylogeny and DNA barcoding of tropical eastern Pacific shallow-water gorgonian octocorals. Marine Biology 161: 1027–1038. <https://doi.org/10.1007/s00227-014-2396-8>

Wirshing HH, Messing CG, Douady CJ, Reed J, Stanhope MJ, Shivji MS (2005) Molecular evidence for multiple lineages in the gorgonian family Plexauridae (Anthozoa: Octocorallia). Marine Biology 147: 497–508. <https://doi.org/10.1007/s00227-005-1592-y>
